# Evolution of human genes encoding cell surface receptors involved in the regulation of appetite: an analysis based on the phylostratigraphic age and divergence indexes

**DOI:** 10.18699/VJGB-23-96

**Published:** 2023-12

**Authors:** E.V. Ignatieva, S.A. Lashin, Z.S. Mustafin, N.A. Kolchanov

**Affiliations:** Institute of Cytology and Genetics of the Siberian Branch of the Russian Academy of Sciences, Novosibirsk, Russia; Institute of Cytology and Genetics of the Siberian Branch of the Russian Academy of Sciences, Novosibirsk, Russia; Institute of Cytology and Genetics of the Siberian Branch of the Russian Academy of Sciences, Novosibirsk, Russia; Institute of Cytology and Genetics of the Siberian Branch of the Russian Academy of Sciences, Novosibirsk, Russia

**Keywords:** regulation of appetite, cell surface receptors, hunger, evolution, phylostratigraphic analysis, gene age, gene variability, регуляция аппетита, рецепторы клеточной поверхности, чувство голода, эволюция, филостратиграфия, возраст гена, изменчивость генов

## Abstract

Genes encoding cell surface receptors make up a significant portion of the human genome (more than a thousand genes) and play an important role in gene networks. Cell surface receptors are transmembrane proteins that interact with molecules (ligands) located outside the cell. This interaction activates signal transduction pathways in the cell. A large number of exogenous ligands of various origins, including drugs, are known for cell surface receptors, which accounts for interest in them from biomedical researchers. Appetite (the desire of the animal organism to consume food) is one of the most primitive instincts that contribute to survival. However, when the supply of nutrients is stable, the mechanism of adaptation to adverse factors acquired in the course of evolution turned out to be excessive, and therefore obesity has become one of the most serious public health problems of the twenty-first century. Pathological human conditions characterized by appetite violations include both hyperphagia, which inevitably leads to obesity, and anorexia nervosa induced by psychosocial stimuli, as well as decreased appetite caused by neurodegeneration, inflammation or cancer. Understanding the evolutionary mechanisms of human diseases, especially those related to lifestyle changes that have occurred over the past 100–200 years, is of fundamental and applied importance. It is also very important to identify relationships between the evolutionary characteristics of genes in gene networks and the resistance of these networks to changes caused by mutations. The aim of the current study is to identify the distinctive features of human genes encoding cell surface receptors involved in appetite regulation using the phylostratigraphic age index (PAI) and divergence index (DI). The values of PAI and DI were analyzed for 64 human genes encoding cell surface receptors, the orthologs of which were involved in the regulation of appetite in model animal species. It turned out that the set of genes under consideration contains an increased number of genes with the same phylostratigraphic age (PAI = 5, the stage of vertebrate divergence), and almost all of these genes (28 out of 31) belong to the superfamily of G-protein coupled receptors. Apparently, the synchronized evolution of such a large group of genes (31 genes out of 64) is associated with the development of the brain as a separate organ in the first vertebrates. When studying the distribution of genes from the same set by DI values, a significant enrichment with genes having a low DIs was revealed: eight genes (GPR26, NPY1R, GHSR, ADIPOR1, DRD1, NPY2R, GPR171, NPBWR1) had extremely low DIs (less than 0.05). Such low DI values indicate that most likely these genes are subjected to stabilizing selection. It was also found that the group of genes with low DIs was enriched with genes that had brain-specific patterns of expression. In particular, GPR26, which had the lowest DI, is in the group of brain-specific genes. Because the endogenous ligand for the GPR26 receptor has not yet been identified, this gene seems to be an extremely interesting object for further theoretical and experimental research. We believe that the features of the genes encoding cell surface receptors we have identified using the evolutionary metrics PAI and DI can be a starting point for further evolutionary analysis of the gene network regulating appetite.

## Abstract (ru)

Гены рецепторов клеточной поверхности составляют существенную долю генома человека (более тысячи генов) и выполняют важную роль в генных сетях. Рецепторы клеточной поверхности – это трансмембранные белки, которые взаимодействуют с различными молекулами (лигандами), находящими- ся во внеклеточном пространстве, что приводит к активации путей сигнальной трансдукции в клетке. Для рецепторов клеточной поверхности известно большое количество экзогенных лигандов различного проис- хождения, включая лекарственные препараты, что и определяет интерес к их исследованию с точки зрения биомедицины. Аппетит (стремление животного организма потреблять пищу) – один из самых примитивных инстинктов, способствующих выживанию. Однако приобретенный в ходе эволюции механизм приспособления к неблагоприятным факторам в условиях стабильного поступления питательных веществ оказался избыточным, в связи с чем ожирение стало одной из самых серьезных проблем общественного здравоохранения в XXI веке. Патологические состояния человека, характеризующиеся нарушениями аппетита, вклю- чают как гиперфагию, неминуемо приводящую к ожирению, так и нервную анорексию, индуцированную психосоциальными стимулами, и снижение аппетита, связанное с воспалительными, нейродегенеративны- ми и онкологическими заболеваниями. Понимание эволюционных механизмов развития болезней челове- ка, особенно связанных с изменениями образа жизни, произошедшими в течение последних 100–200 лет, имеет как фундаментальное, так и прикладное значение. Особенно важно установить взаимосвязи между эволюционными характеристиками генов в генных сетях и устойчивостью этих сетей к изменениям, вызван- ным мутациями. Цель данной работы – выявление особенностей эволюции генов рецепторов клеточной поверхности человека, участвующих в регуляции аппетита, с использованием филостратиграфического ин- декса PAI (phylostratigraphic age index) и индекса эволюционной изменчивости DI (divergence index). Были проанализированы индексы PAI и DI для 64 генов человека, кодирующих рецепторы клеточной поверхности, ортологи которых участвовали в регуляции аппетита у модельных видов животных. Оказалось, что в рас- сматриваемом наборе генов содержится повышенное количество генов, имеющих одинаковый филостра- тиграфический возраст (PAI = 5, этап дивергенции позвоночных), и почти все эти гены (28 из 31) относятся к суперсемейству рецепторов, сопряженных с G-белком. По-видимому, синхронизированное эволюциониро- вание такой многочисленной группы генов (31 из 64 генов) связано с формированием у первых позвоноч- ных мозга как отдельного органа. При исследовании распределения генов из этого же набора по значениям индексов DI была выявлена существенная обогащенность генами с низким DI. При этом восемь генов (GPR26, NPY1R, GHSR, ADIPOR1, DRD1, NPY2R, GPR171, NPBWR1) характеризовались экстремально низким значением DI (менее 0.05), что указывает на существенную их подверженность стабилизирующему отбору. Обнаружено также, что группа генов с низким DI обогащена генами, тканеспецифически экспрессирующимися в мозге. В частности, к группе генов, тканеспецифически экспрессирующихся в мозге, относится GPR26, имеющий самое низкое значение DI. Ввиду того, что эндогенный лиганд для рецептора GPR26 пока не выявлен, этот ген представляется чрезвычайно интересным объектом для дальнейшего теоретического и экспериментально- го исследования. Выявленные нами особенности распределения генов рецепторов клеточной поверхности по эволюционным индексам PAI и DI являются отправной точкой для дальнейшего анализа эволюционных характеристик генной сети регуляции аппетита в целом.

## Introduction

Appetite (the desire of the animal organism to consume food) is a physiological mechanism (feeling) that regulates the intake of nutrients. The desire to consume food is one of the most primitive instincts that contribute to survival. This instinct has been formed over millions of years of evolution of living beings and has provided powerful mechanisms for adaptation and response to periods of nutrient shortage (Yeo, Heisler, 2012). The ability to consume excessive amounts of food during periods of its availability significantly affected the survival of individuals both in human populations and in populations of other animal species.

With the development of human civilization, the human populations living in developed countries faced the problem of adaptation to the abundance of food combined with a decrease in physical activity, making obesity one of the most serious public health problems of the twenty-first century (Kaidar- Person et al., 2011). Thus, the mechanism of adaptation to unfavourable factors acquired during evolution in conditions of stable nutrient supply turned out to be excessive (Yeo, Heisler, 2012)

In humans and other animal species, the physiological system that regulates appetite functions with the participation of protein products of genes expressed both in the brain (Olszewski et al., 2008) and in peripheral organs and tissues: stomach, intestine, pancreas, adipose tissue. Neurons involved in the regulation of the motivational drive to obtain food are located in different parts of the brain (hypothalamic nuclei, amygdala, dorsal raphe nucleus, nuclei of the solitary tract, ventral tegmental area, prefrontal cortex, etc.). They integrate signals received from the sensory organs (olfactory, visual, taste sensations) as well as various interoceptive and humoral signals and control search for food and food consumption (Yeo, Heisler, 2012; Tremblay, Bellisle, 2015; Heisler, Lam, 2017).

Appetite can be induced by energy and nutrient shortages (in this case it is referred to by the term homeostatic appetite). However, even in the absence of apparent homeostatic needs, factors such as the sight, smell and taste of food, environmental cues, and the anticipation of new sensations that arise from eating can stimulate eating behavior, i. e. non-homeostatic appetite. The neuronal systems controlling homeostatic and non-homeostatic appetite function in close cooperation (Ahn et al., 2022).

Neurons of the arcuate nucleus of the hypothalamus secreting neuropeptide Y (NPY), agouti-like protein (AgRP), and alpha-melanocyte stimulating hormone (α-MSH), which is generated as a proteolytic cleavage product from proopiomelanocortin (POMC) by prohormone convertases (PCSK1 and PCSK2), are central to the systems regulating both homeostatic and non-homeostatic appetite (Yeo, Heisler, 2012). The activity of neurons located in the arcuate nucleus is controlled by hormones (leptin, insulin, ghrelin, polypeptide YY (PYY), glucocorticoids, adrenocorticotropin, corticotropin-releasing hormone), neurotransmitters (serotonin, dopamine, adrenaline, GABA), and neurotrophic factors (BDNF, etc.) as well (Maniam, Morris, 2012; Yeo, Heisler, 2012; Heisler, Lam, 2017).

Human pathological conditions characterized by appetite disorders are known. A pathological increase in body weight (obesity) can be caused by such a condition as hyperphagia (bulimia). A catastrophic decrease in appetite is seen in anorexia nervosa, which is extremely dangerous and increases the risk of death in young people tenfold (Fichter, Quadflieg, 2016). Reduced appetite can accompany chronic inflammatory and autoimmune processes, cancer and neurodegenerative diseases (Grossberg et al., 2010). In this context, any new knowledge about the system of genes regulating appetite is of particular importance.

Previously, we performed a functional analysis of genes involved in the regulation of appetite and body weight (Ignatieva et al., 2014, 2016). When analyzing a set of 105 genes involved in appetite regulation, a statistically significant over-enrichment of genes specifically expressed in the brain was found. It was also revealed that a substantial proportion of genes (~45 %) in this set were genes encoding cell surface receptors. Many of these receptors belonged to the superfamily of G-protein-coupled receptors (GPCRs).

The GPCRs superfamily includes proteins that have a similar structure (all of them contain 7 transmembrane domains). These proteins can be found on the cell membranes of almost all eukaryotes (New, Wong, 1998; Yang et al., 2021). Analysis of the DNA sequence of the human genome made it possible to predict about 800 genes encoding proteins of this superfamily (including 388 genes encoding olfactory receptors) (Bjarnadóttir et al., 2006). GPCRs mediate the response of cells to extracellular signaling molecules of different nature – proteins, peptides, low molecular weight substances (odorous and taste stimuli, hormones), as well as light-sensitive compounds. In turn, these receptors activate signal transduction pathways in cells, providing fundamental physiological processes (vision, perception of taste and olfactory signals, neuronal functioning, endocrine regulation and reproduction processes) (Katritch et al., 2013). Some of the best known receptors from the GPCR superfamily, which we have previously classified as appetite-regulating genes (Igatieva et al., 2016), include, for example, GHSR (growth hormone secretagogue receptor), MC3R (melanocortin 3 receptor), MC4R (melanocortin 4 receptor), CCKAR (cholecystokinin A receptor), CCKBR (cholecystokinin B receptor) and GCGR (glucagon).

Understanding the evolutionary mechanisms of human diseases, especially those associated with lifestyle changes that have occurred over the last 100–200 years (and the above-mentioned diseases associated with appetite dysregulation are just such diseases), is of great fundamental and applied significance. It is also very important to find interdependence between the evolutionary characteristics of genes in gene networks and the resistance of these networks to disruptions of genes themselves (through mutations) and to alterations in gene expression patterns caused by genetic variability of regulatory regions. Phylogenetic and population analysis of genes and gene networks involved in the relevant biological processes may be useful in developing new scenarios for personalized prevention and targeted drug therapy of diseases.

The aim of this work was to identify the evolutionary features of human cell surface receptor genes involved in appetite regulation using phylostratigraphic age index (PAI) and divergence index (DI). To achieve this goal, at the first stage, a set of human receptor genes the orthologues of which were involved in appetite regulation in model organisms was formed based on the analysis of scientific publications. Next, the distributions of human genes according to PAI and DI values were examined. The characteristic features of these distributions were identified by comparison with the distributions obtained for all human protein-coding genes, as well as for genes encoding GPCRs.

## Materials and methods

Collecting the list of genes involved in appetite regulation and encoding cell surface receptors. The list of genes was taken from (Ignatieva et al., 2016) and expanded based on a PubMed search (https://pubmed.ncbi.nlm.nih.gov/) using the keywords listed in Supplementary Material 11. Only genes from experimental studies were considered; reviews were excluded. In most studies, the role of genes in the regulation of food consumption was established using model organisms (mice, rats, etc.). Therefore, the list of human genes controlling appetite included orthologues of those genes that were identified in experiments on other animal species. Indication that the product of a gene is a cell surface receptor was obtained from the text field “Summary” of the EntrezGene database (https://www.ncbi.nlm.nih.gov/gene).


Supplementary Materials are available in the online version of the paper: https://vavilov.elpub.ru/jour/manager/files/Suppl_Ignatieva_Engl_27_7.pdf


Control sets of genes. The human gene sets listed in Table 1 were also used in the analyses. The list of human genes encoding receptors and controlling appetite was named Receptors_64.

**Table 1.**
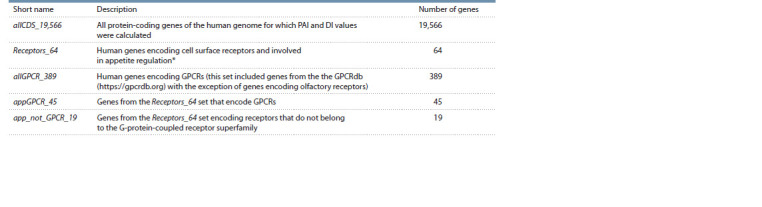
Gene sets for which the distributions of PAI and DI values were analysed * This set includes human genes orthologous to genes of other animal species, the role of which in appetite regulation has been studied experimentally.

The set containing all human protein-coding genes (allCDS_19,566) included 19,566 protein-coding genes for which PAI and DI values were identified.

The set containing human genes encoding GPCRs (allGPCR_389) was formed on the basis of the GPCRdb (https://gpcrdb.org) (Pandy-Szekeres et al., 2023). Genes encoding olfactory receptors were not included in this set because the set of appetite-controlling cell surface receptor genes (Receptors_64) did not contain genes encoding receptors of this type.

The set containing genes encoding GPCRs that control appetite (appGPCR_45) was obtained by the intersection of two sets – Receptors_64 and allGPCR_389.

Analysis of the evolutionary characteristics of genes. The analysis was performed using PAI (phylostratigraphic age index) and DI (divergence index).

Phylostratigraphic age index (PAI) shows to what extent the taxon reflecting the age of the gene is distant from the root of the phylogenetic tree. The taxon reflecting the age of the gene is understood as the taxon at which the divergence of the studied species from the most distant related taxon, in which the orthologue of the gene in question was found, occurred (Table 2). The greater the PAI value of the gene under study, the younger this gene is supposed to be. PAI values were calculated in the Orthoscape tool based on the KEGG Orthology service as described in (Mustafin et al., 2021). We used PAI values calculated at a similarity level of 0.5.

**Table 2.**
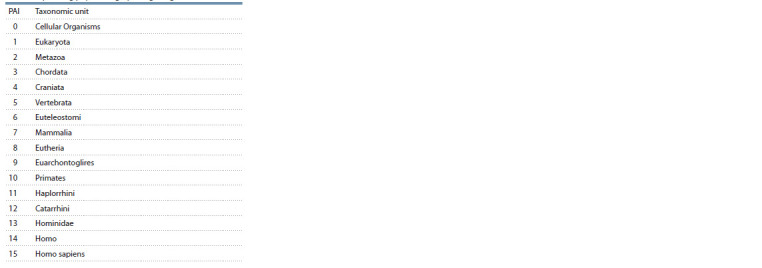
PAI values and taxonomic units dating the corresponding phylostratigraphic age of genes

Divergence index (DI) is an index of evolutionary variability of a gene. DI is calculated based on the dN/dS ratio, where dN is the proportion of nonsynonymous substitutions in DNA sequences of the studied gene and its orthologue; dS is the proportion of synonymous substitutions. The value of this index was calculated based on the comparison of human genes with genes of closely related organisms from the Hominidae family, as described in (Mustafin et al., 2021). Thus, DI can be determined only for protein-coding genes and indicates the type of selection acting on the gene. DI value in the range from 0 to 1 shows that the gene is subjected to stabilizing selection, 1 – to neutral evolution, and more than 1 – to driving selection.

Analysis of tissue-specific characteristics of genes. We used the TSEA tool to find overrepresented groups of genes that had tissue-specific expression patterns identified for a certain organ or tissue (Wells et al., 2015). The TSEA tool (http://genetics.wustl.edu/jdlab/tsea/) uses data on tissue-specific gene expression patterns detected in 25 different human organs and tissues. The TSEA tool identifies groups of tissuespecific genes in the analyzed list of genes, the size of which significantly exceeds the expected one for random reasons. The TSEA tool uses data on specificity indices (SI) of gene expression products and their corresponding p-values (pSI). These values were calculated for each organ or tissue and for each transcript based on the analysis of data obtained by whole transcriptome profiling (GTEx Consortium, 2015). If the pSI value was <0.01, the transcript was considered as tissue-specific for a given tissue.

Statistical analysis. The significance of differences between the observed and expected numbers of genes in subgroups was evaluated using the Chi-Square test.

## Results

Genes encoding cell surface receptors and their functional characteristics

As a result of queries to PubMed, experimental data on genes of model organisms (mice, rats, etc.) involved in the regulation of food intake were found. Using this information, as well as information from EntrezGene, we found 64 human genes orthologous to genes identified in model organisms and encoding cell surface receptors (in Table 1 this set of genes is presented as Receptors_64). See Supplementary Material 2 for the full list of genes

**Fig. 1.**
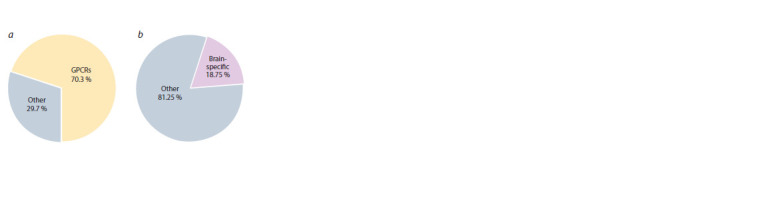
Functional characteristics of human genes encoding cell surface receptors and involved in appetite regulation (genes from the Receptors_ 64 set). a, The proportion of genes encoding GPCRs; b, the proportion of genes that have brain-specific expression pattern (tissue-specific genes were identified using the TSEA tool).

We compared these 64 genes with those accumulated in GPCRdb and found that 45 (70.3 %) out of 64 genes encoded G-protein-coupled receptors (Fig. 1, a). As is shown in Table 1, this subset of genes encoding receptors from the GPCR superfamily was named appGPCR_45 (Supplementary Material 2 contains data on whether the gene belongs to the GPCR superfamily). The remaining 19 genes (29.7 %) encoded receptors from the other superfamilies (this subset is represented in Table 1 as app_not_GPCR_19).

Analysis of the gene list using the TSEA tool (Wells et al., 2015) revealed that the Receptors_64 set was enriched in genes that have brain-specific expression pattern. Approximately one fifth of the genes (12 genes, or 18.75 %) fall into this category (see Fig. 1, b, Supplementary Material 3).

Analysis of evolutionary characteristics

**Fig. 2.**
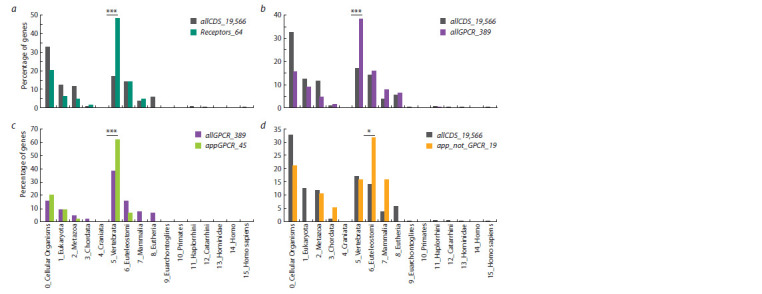
Distributions by PAI values obtained for the sets of human protein-coding genes presented in Table 1. a: all human protein-coding genes (allCDS_19,566) as a control set and the human appetite-regulating genes encoding receptors (Receptors_64); b: all human protein-coding genes (allCDS_19,566) as a control set and genes encoding GPCRs (allGPCR_389); c: genes encoding GPCRs as a control set (allGPCR_389) and genes encoding GPCRs controling appetite (appGPCR_45); d: all human protein-coding genes (allCDS_19,566) as a control set and genes controlling appetite but not belonging to the GPCRs superfamily (app_not_GPCR_19). PAI values were calculated at a threshold of 0.5 for the level of similarity between the DNA sequences of the orthologous genes. Asterisks indicate differences between the number of genes with a PAI equal to 5 (the stage of vertebrate divergence) (a–c) or a PAI equal to 6 (the stage of euteleostomi divergence) (d) and their expected numbers calculated based on the distributions in the control sets. *** p <0.001, * p <0.05. See Supplementary Materials 5–8.

Phylostratigraphic age of genes (PAI-based analysis). At the first step, we have analyzed the distribution of PAI values for all human protein-coding genes (the allCDS_19,556 set). PAI values were found to be unevenly distributed (Fig. 2, a). One third of all genes (33 %) had a PAI equal to zero (cellular organisms, the root of the phylogenetic tree), and the proportions of genes that had PAI values equal to 5 (the stage of vertebrate divergence) and 6 (the stage of euteleostomi divergence) were 17 % and 14 %, respectively. When we examined the distribution of PAI values for a set of human genes encoding cell surface receptors and involved in appetite regulation (the Receptors_64 set, Supplementary Material 4), we found that 31 genes out of 64 (i. e. 48 %) had PAI values equal to 5 (the stage of vertebrate divergence) (see Fig. 2, a). And this number was significantly higher (p < 0.001) than the expected number (10.898) calculated based on the distribution obtained for the allCDS_19,556 gene set (see Fig. 2, a, Supplementary Material 5).

As noted above, a large proportion of genes from the Receptors_64 set (45 genes out of 64) encode GPCRs (see Fig. 1, a). To find out whether the evolutionary features of genes from the Receptors_64 set are caused by the features of genes from the GPCRs superfamily, we analysed the distribution of PAI values for a set of 389 human genes encoding GPCRs represented in the GPCRdb database (https://gpcrdb.org) (allGPCR_389 set). This distribution was also found to be different from the distribution obtained for all human protein-coding genes (see Fig. 2, b). The number of genes in the allGPCR_389 set that had PAI values equal to 5 (the stage of vertebrate divergence) was 39 % (150 genes out of 389) and it was significantly higher than the expected number calculated based on the proportion of this group of genes in the allCDS_19,566 set (Supplementary Material 6).

Next, we compared the distribution of PAI values for 45 genes encoding GPCRs and regulating appetite (the appGPCR_45 set) with the distribution for the allGPCR_389 set (see Fig. 2, c). In the group of genes from the appGPCR_45 set, 28 genes were found to have a PAI equal to 5 (the stage of vertebrate divergence) (i. e. 64 %), which was significantly higher than the expected number (17.35) calculated based on the proportion of this group of genes in the allGPCR_389 set (Supplementary Material 7).

As mentioned earlier, 19 receptor genes controlling appetite did not belong to the G-protein-coupled receptor superfamily (the app_not_GPCR_19 set). When the distribution over PAI values for this group of genes was examined, it was also found to differ from the distribution over PAI values for all human protein-coding genes (see Fig. 2, d). However, in this case, a significant (p < 0.05) excess over the expected number of genes with a PAI equal to 6 (the stage of euteleostomi divergence) was observed. The observed number in the app_not_GPCR_19 set was six genes out of 19 (32 %), whereas in the allCDS_19,566 set, PAI value equal to 6 was detected for 2,769 genes (14 %). Thus, the expected number of genes with PAI equal to 6 in the app_not_GPCR_19 set was 2.69 (Supplementary Material 8).

**Fig. 3.**
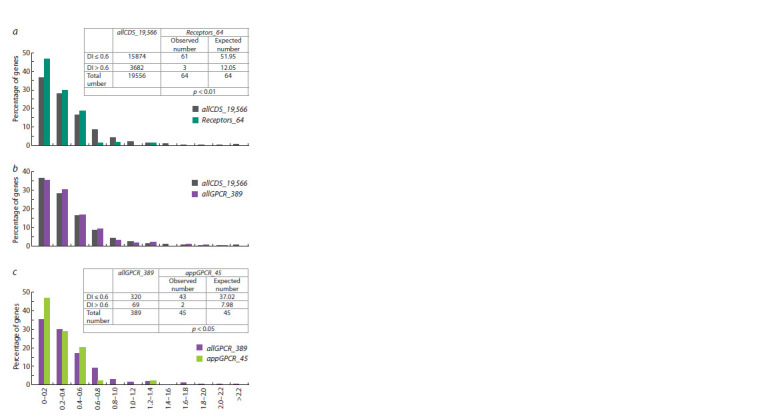
Distributions of genes from the sets presented in Table 1 according to the DI index. a, All human protein-coding genes (allCDS_19,566) as a control set and and the human appetite-regulating genes encoding receptors (Receptors_64). The observed and expected total number of genes with DI ≤ 0.6 and DI > 0.6 are presented in the table above the graph; the calculation of the expected number is given in Supplementary Material 10. b, All human proteincoding genes (allCDS_19,566) as a control set and genes encoding GPCRs (allGPCR_389). c, Genes encoding GPCRs (allGPCR_389) as a control set and genes encoding GPCRs controling appetite (appGPCR_45). The observed and expected total number of genes with DI ≤ 0.6 and DI > 0.6 are presented in the table above the graph; the calculation of the expected number is given in Supplementary Material 11.

Evolutionary variability of genes (DI-based analysis). The analysis of the distribution of genes from the Receptors_64 set according to DI values (Fig. 3, a, Supplementary Material 9) showed that 47 % of genes (30 out of 64) had DI < 0.2, most genes (63 out of 64, i. e. ~98 %) had DI < 1, and only one gene (QRFPR) had DI > 1, indicating that most of the genes are subjected to stabilizing selection.

Comparison of the distribution of genes from the Receptors_64 set by DI values with the distribution obtained for all human protein-coding genes (allCDS_19,566 set) showed that the Receptors_64 set is characterised by an increased content of genes with low DI values (see Fig. 3, a). The majority of genes from the Receptors_64 set (61 genes out of 64, i. e. 95 %) had DI < 0.6. And this number was significantly (p < 0.01) higher than the expected number (51.95) calculated using the distribution obtained for all human protein-coding genes (see Fig. 3, a, Supplementary Material 10).

When comparing the distribution over DI values for a set of all receptors from the GPCRs superfamily (allGPCR_389) with the distribution for all protein-coding genes (allCDS_19,566), no significant differences were found (see Fig. 3, b).

Comparison of the distribution over DI values for the appGPCR_45 set with the distribution for all receptors from the GPCRs superfamily (allGPCR_389) showed that the number of genes with low DI (DI ≤ 0.6) in the appGPCR_45 set (42 genes) was significantly (p < 0.05) higher than the expected number of genes (37.018) calculated from the DI distribution for all genes encoding GPCRs (see Fig. 3, c, Sup- plementary Material 11).

**Fig. 4.**
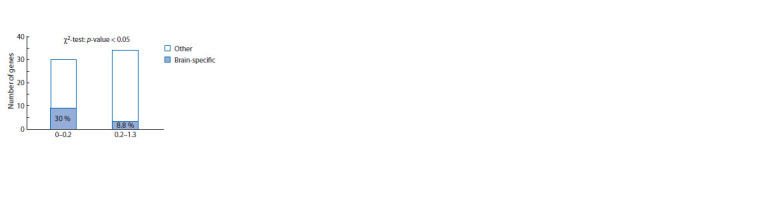
Distribution of genes from the Receptors_64 set by DI values. Shown are the proportions of genes that have brain-specific expression patterns according to the TSEA tool. The observed number of brain-specific genes differ from the expected number, * p < 0.05. (The numbers of genes in four subgroups are given in Supplementary Material 12.)

As indicated above, approximately one-fifth (18.75 %) of genes from the Receptors_64 set are brain-specific. We have determined the content of genes that had brain-specific expression patterns in two subgroups of genes: (1) a subgroup of genes with low DI (DI ≤ 0.2); (2) a subgroup including all other genes (they had DI values between 0.2 and 1.3). It turned out that the number of brain-specific genes in these subgroups differs significantly from the expected number calculated based on random distribution: in the subgroup of genes with low DI, the content of brain-specific genes was increased (Fig. 4, Supplementary Material 12).

## Discussion

Cell surface receptor genes constitute a substantial proportion (more than a thousand genes) of the human genome (Bausch- Fluck et al., 2018). The interest in the study of cell surface receptors is due to their important role in the cell. These transmembrane proteins interact with various molecules (ligands) located in the extracellular space and activate signal transduction pathways in the cell (Bausch-Fluck et al., 2018; Yang et al., 2021). A lot of substances and biochemical compounds (in particular, drugs) that affect the activity of cell surface receptors (so-called agonists and antagonists) are known. Therefore, cell surface receptors are also of great interest from a biomedical point of view – for example, these proteins are targets for 66% of drugs registered in the DrugBank database (Bausch-Fluck et al., 2018).

This paper presents a set of 64 human genes encoding cell surface receptors, the orthologs of which are involved in food intake regulation in model organisms. The data are highly reliable, because this gene set was created on the basis of manual analysis of scientific publications. Finding such an impressive number of receptor genes involved in appetite regulation fits well with the idea of the complex nature of food motivation. As mentioned above, appetite can satisfy both the basic needs of the organism for food (homeostatic appetite, which provides compensation of energy expenditure) and the needs for sensations associated with food (non-homeostatic appetite, aimed at obtaining positive emotions) (Johnson, 2013; Rebello, Greenway, 2016; Ahn et al., 2022). It is also known that food motivation can be adjusted depending on the life situation or psycho-emotional state of an individual (fright, depression, boredom, chronic stress, for animals – threat from predators, territory protection, mating behavior, etc.) (Lindén et al., 1987; Braden et al., 2018, 2023; Hadjieconomou et al., 2020; Siegal et al., 2022). Such correction of food motivation is performed because the brain processes information received from the sensory organs and integrates it with signals about the state of various physiological systems of the body (Tomé et al., 2009; Holtmann, Talley, 2014; Spetter et al., 2014; Tremblay, Bellisle, 2015). And this process involves nerve cells with diverse specialization expressing a wide range of receptors on their surface (Yeo, Heisler, 2012; Heisler, Lam, 2017).

Examination of the distributions of genes by PAI values revealed that: (1) the Receptors_64 set has a significantly increased content of genes with the same phylostratigraphic age (PAI = 5, the stage of vertebrate divergence) than all proteincoding genes; (2) the subset of genes that encode GPCRs and are involved in appetite regulation (appGPCR_45) also contains an increased number of genes with the same phylostratigraphic age (PAI = 5, the stage of vertebrate divergence) than what would be expected based on the distribution of PAI values for all genes encoding GPCRs.

Thus, we found that the gene set composed of genes encoding cell surface receptors controlling appetite contains an increased number of genes with the same phylostratigraphic age (PAI = 5, the stage of vertebrate divergence). Apparently, the synchronised evolution of such a large group of genes (31 genes have PAIs equal to five) is associated with the formation of the brain as a separate organ in the first vertebrates (Sarnat, Netsky, 2002). It is noteworthy that almost all of these genes with PAI equal to five (28 out of 31) encode GPCRs, which agrees well with the fact that receptors of this superfamily are involved in processing signals from sensory organs, as well as signals transmitted by hormones and neuromediators (Pandy-Szekeres et al., 2023). Thus, the group of genes encoding GPCRs with a PAI of 5 (the stage of vertebrate divergence) includes, in particular, genes encoding receptors for neuropeptide Y (NPY1R, NPY2R, NPY4R, NPY5R) and alpha-melanocyte stimulating hormone (MC3R and MC4R). Neuropeptide Y and alpha-melanocyte stimulating hormone are signalling molecules secreted by neurons of the arcuate nucleus of the hypothalamus, a brain structure that acts as a central regulator of feeding behaviour (Yeo, Heisler, 2012; Heisler, Lam, 2017).

Another peculiarity was revealed for a subset of genes involved in appetite regulation but not encoding GPCRs (app_not_GPCR_19): it contains an increased number of genes with PAI equal to 6 (the stage of euteleostomi divergence). Notably, four genes from this group encode receptors involved in the regulation of immunity. These are GHR and LEPR encoding proteins from the type I cytokine receptor family and TLR2 and TLR4 encoding proteins from the Tolllike receptor family.

The PAI-based analysis has shown that the so-called “ancient” genes (i. e., genes with PAI equal to 0 (cellular organisms, the root of the taxonomic tree)) are also involved in the regulation of food intake. This group includes, for example, genes encoding (1) the insulin receptor (INSR), which, in particular, regulates secretion of neuropeptide Y and alphamelanocyte stimulating hormone by neurons of the arcuate nucleus of the hypothalamus (Leibowitz, Wortley, 2004), and (2) NTRK2, the receptor for BDNF (brain-derived neurotrophic factor), which mediates the anorexigenic effects of BDNF produced in the paraventricular nucleus of the hypothalamus (An et al., 2015; Chu et al., 2023). Both genes are expressed in different tissues and organs (Escandón et al., 1994; Federici et al., 1997), indicating that at early stages of evolution, ancestral forms of INSR and NTRK2 could be involved in the regulation of various biological processes and joined the system of genes regulating food intake at the evolutionary stage corresponding to the formation of specialised brain structures

When considering PAI values, a group of relatively “young” genes was identified (PAI values of 6 and 7, the stages of euteleostomi and mammalia divergence). Five genes from this group encode receptors relevant to the immune system: these are the four genes mentioned earlier (GHR, LEPR, TLR2, and TLR4), as well as IL1R1. The detection of these genes in a group of relatively “young” genes is in good agreement with the known data on the adaptive immunity system having begun to form relatively recently in the course of evolution (Ward, Rosenthal, 2014).

When examining the distribution of genes from the Receptors_ 64 set by DI values, a significant enrichment of this group with genes subjected to stabilizing selection was revealed. It turned out that the subgroup of appetite-regulating genes encoding GPCRs (appGPCR_45) also contained an increased number of genes with low DI values.

Eight genes had the lowest DI values (DI < 0.05): GPR26, NPY1R, GHSR, ADIPOR1, DRD1, NPY2R, GPR171, NPBWR1 (see Supplementary Material 9). Moreover, seven of these eight genes (except ADIPOR1) encode proteins from the GPCRs superfamily

An extremely low DI value (<0.005) was found for the GPR26 gene. GPR26 encodes a receptor from the GPCRs superfamily, the endogenous ligand of which has not yet been identified. Targeted inactivation of GPR26 in mice causes hyperphagia leading to early onset of diet-induced obesity (Chen et al., 2012). In addition, according to behavioural tests, Gpr26-deficient mice display increased anxiety- and depression-like behavior, and prefer ethanol to a greater extent than mice with normal genotype (Zhang et al., 2011). According to the TSEA tool, GPR26 has brain-specific expression pattern. In humans, GPR26 is expressed in the amygdala, hippocampus, and thalamus (Jones et al., 2007). The function of the GPR26 gene is evolutionarily conserved. In C. elegans, the Y5H2B gene with similarity to GPR26 was found. Ashrafi K. and co-workers used RNA-mediated interference to disrupt the expression of genes and found that Y5H2B increases fat content (Ashrafi et al., 2003). The functions of other genes that had extremely low DI values (<0.05) are described in Supplementary Material 13.

Only one gene (QRFPR) among the genes from the Receptors_ 64 set had a DI > 1 (see Supplementary Material 9), indicating that this gene is probably subjected to driving selection. QRFPR encodes the receptor for the orexigenic neuropeptide QRFP (pyroglutamylated RFamide peptide) (Cook et al., 2022). According to EntrezGene and UniProt databases, human QRFPR is expressed in different parts of the brain and in peripheral tissues (heart, kidney, stomach, testes, and thyroid gland). The mouse, rat, and hamster genomes are known to contain at least two genes encoding the neuropeptide receptor QRFP (Cook et al., 2022). No data like that are available for the human genome; however, it can be assumed that human QRFPR is not subjected to stabilizing selection, since the human genome also contains more than one gene encoding proteins with QRFPR-like activity.

We also found that the group of genes with lowDI, i. e. most likely to be subject to stabilizing selection, is enriched in genes that have brain-specific expression patterns. This result agrees well with the finding made by G. Dumas et al. who examined the set of almost all human protein-coding genes (N = 11,667) and revealed that genes encoding brain-related proteins are among the most strongly conserved protein-coding genes in the human genome (Dumas et al., 2021). Among the genes that have low DI and brain-specific expression pattern, the previously mentioned GPR26 gene was found. Due to the fact that this gene has an extremely low DI and its endogenous ligand is still unknown (Chen et al., 2012), GPR26 seems to be an extremely interesting object for further theoretical and experimental studies.

## Conclusion

In this paper, we analyzed the distributions of PAI and DI values for a group of human cell surface receptor genes, the orthologues of which were involved in appetite regulation in model organisms. It was found that the gene set under consideration contains an increased number of genes with the same phylostratigraphic age (PAI = 5, the stage of vertebrate divergence), which is apparently associated with the formation of the brain as a separate organ in the first vertebrates. A significant enrichment of this group of genes in genes with low DI values was also revealed, indicating a significant susceptibility of these genes to stabilizing selection. At the same time, the group of genes with low DI is enriched with genes that have brain-specific expression pattern. The characteristic features of the cell surface receptor genes distribution according to the evolutionary indices PAI and DI revealed in this study are a starting point for further analyses of the evolutionary characteristics of the entire gene network controlling appetite

## Conflict of interest

The authors declare no conflict of interest.

## References

Ahn B.H., Kim M., Kim S.Y. Brain circuits for promoting homeostatic
and non-homeostatic appetites. Exp. Mol. Med. 2022;54(4):349-
357. DOI 10.1038/s12276-022-00758-4

An J.J., Liao G.Y., Kinney C.E., Sahibzada N., Xu B. Discrete BDNF
neurons in the paraventricular hypothalamus control feeding and
energy expenditure. Cell Metab. 2015;22(1):175-188. DOI 10.1016/
j.cmet.2015.05.008

Ashrafi K., Chang F.Y., Watts J.L., Fraser A.G., Kamath R.S., Ahringer
J., Ruvkun G. Genome-wide RNAi analysis of Caenorhabditis
elegans fat regulatory genes. Nature. 2003;421(6920):268-272. DOI
10.1038/nature01279

Bausch-Fluck D., Goldmann U., Müller S., van Oostrum M., Müller M.,
Schubert O.T., Wollscheid B. The in silico human surfaceome. Proc.
Natl. Acad. Sci. USA. 2018;115(46):E10988-E10997. DOI 10.1073/
pnas.1808790115

Bjarnadóttir T.K., Gloriam D.E., Hellstrand S.H., Kristiansson H.,
Fredriksson R., Schiöth H.B. Comprehensive repertoire and phylogenetic
analysis of the G protein-coupled receptors in human
and mouse. Genomics. 2006;88(3):263-273. DOI 10.1016/j.ygeno.
2006.04.001

Braden A., Musher-Eizenman D., Watford T., Emley E. Eating when
depressed, anxious, bored, or happy: are emotional eating types associated
with unique psychological and physical health correlates?
Appetite. 2018;125:410-417. DOI 10.1016/j.appet.2018.02.022

Braden A., Barnhart W.R., Kalantzis M., Redondo R., Dauber A.,
Anderson L., Tilstra-Ferrell E.L. Eating when depressed, anxious,
bored, or happy: an examination in treatment-seeking adults with
overweight/obesity. Appetite. 2023;184:106510. DOI 10.1016/
j.appet.2023.106510

Chen D., Liu X., Zhang W., Shi Y. Targeted inactivation of GPR26
leads to hyperphagia and adiposity by activating AMPK in the hypothalamus.
PLoS One. 2012;7(7):e40764. DOI 10.1371/journal.
pone.0040764

Chu P., Guo W., You H., Lu B. Regulation of satiety by Bdnf-e2-expressing
neurons through TrkB activation in ventromedial hypothalamus.
Biomolecules. 2023;13(5):822. DOI 10.3390/biom13050822

Cook C., Nunn N., Worth A.A., Bechtold D.A., Suter T., Gackeheimer
S., Foltz L., Emmerson P.J., Statnick M.A., Luckman S.M. The
hypothalamic RFamide, QRFP, increases feeding and locomotor activity:
the role of Gpr103 and orexin receptors. PLoS One. 2022;
17(10):e0275604. DOI 10.1371/journal.pone.0275604

Dumas G., Malesys S., Bourgeron T. Systematic detection of brain protein-
coding genes under positive selection during primate evolution
and their roles in cognition. Genome Res. 2021;31(3):484-496. DOI
10.1101/gr.262113.120

Escandón E., Soppet D., Rosenthal A., Mendoza-Ramírez J.L., Szönyi
E., Burton L.E., Henderson C.E., Parada L.F., Nikolics K. Regulation
of neurotrophin receptor expression during embryonic and
postnatal development. J. Neurosci. 1994;14(4):2054-2068. DOI
10.1523/JNEUROSCI.14-04-02054.1994>

Federici M., Porzio O., Zucaro L., Fusco A., Borboni P., Lauro D.,
Sesti G. Distribution of insulin/insulin-like growth factor-I hybrid
receptors in human tissues. Mol. Cell. Endocrinol. 1997;129(2):
121-126. DOI 10.1016/s0303-7207(97)04050-1

Fichter M.M., Quadflieg N. Mortality in eating disorders – results of
a large prospective clinical longitudinal study. Int. J. Eat. Disord.
2016;49(4):391-401. DOI 10.1002/eat.22501

Grossberg A.J., Scarlett J.M., Marks D.L. Hypothalamic mechanisms
in cachexia. Physiol. Behav. 2010;100(5):478-489. DOI 10.1016/
j.physbeh.2010.03.011

GTEx Consortium. Human genomics. The Genotype-Tissue Expression
(GTEx) pilot analysis: multitissue gene regulation in humans.
Science. 2015;348(6235):648-660. DOI 10.1126/science.1262110

Hadjieconomou D., King G., Gaspar P., Mineo A., Blackie L., Ameku
T., Studd C., de Mendoza A., Diao F., White B.H., Brown A.E.X.,
Plaçais P.Y., Préat T., Miguel-Aliaga I. Enteric neurons increase
maternal food intake during reproduction. Nature. 2020;587(7834):
455-459. DOI 10.1038/s41586-020-2866-8

Heisler L.K., Lam D.D. An appetite for life: brain regulation of hunger
and satiety. Curr. Opin. Pharmacol. 2017;37:100-106. DOI
10.1016/j.coph.2017.09.002

Holtmann G., Talley N.J. The stomach-brain axis. Best Pract. Res. Clin.
Gastroenterol. 2014;28(6):967-979. DOI 10.1016/j.bpg.2014.10.001

Ignatieva E.V., Afonnikov D.A., Rogaev E.I., Kolchanov N.A. Human
genes controlling feeding behavior or body mass and their functional
and genomic characteristics: a review. Vavilovskii Zhurnal
Genetiki i Selektsii = Vavilov Journal of Genetics and Breeding.
2014;18(4/2):867-875 (in Russian)

Ignatieva E.V., Afonnikov D.A., Saik O.V., Rogaev E.I., Kolchanov
N.A. A compendium of human genes regulating feeding behavior
and body weight, its functional characterization and identification
of GWAS genes involved in brain-specific PPI network. BMC
Genet. 2016;17(Suppl.3):158. DOI 10.1186/s12863-016-0466-2

Johnson A.W. Eating beyond metabolic need: how environmental cues
influence feeding behavior. Trends Neurosci. 2013;36(2):101-109.
DOI 10.1016/j.tins.2013.01.002

Jones P.G., Nawoschik S.P., Sreekumar K., Uveges A.J., Tseng E.,
Zhang L., Johnson J., He L., Paulsen J.E., Bates B., Pausch M.H.
Tissue distribution and functional analyses of the constitutively active
orphan G protein coupled receptors, GPR26 and GPR78. Biochim.
Biophys. Acta. 2007;1770(6):890-901. DOI 10.1016/j.bbagen.
2007.01.013

Kaidar-Person O., Bar-Sela G., Person B. The two major epidemics
of the twenty-first century: obesity and cancer. Obes. Surg. 2011;
21(11):1792-1797. DOI 10.1007/s11695-011-0490-2

Katritch V., Cherezov V., Stevens R.C. Structure-function of the G protein-
coupled receptor superfamily. Annu. Rev. Pharmacol. Toxicol.
2013;53:531-556. DOI 10.1146/annurev-pharmtox-032112-135923

Leibowitz S.F., Wortley K.E. Hypothalamic control of energy balance:
different peptides, different functions. Peptides. 2004;25(3):473-
504. DOI 10.1016/j.peptides.2004.02.006

Lindén A., Hansen S., Bednar I., Forsberg G., Södersten P., Uvnäs-Moberg
K. Sexual activity increases plasma concentrations of cholecystokinin
octapeptide and offsets hunger in male rats. J. Endocrinol.
1987;115(1):91-95. DOI 10.1677/joe.0.1150091

Maniam J., Morris M.J. The link between stress and feeding behaviour.
Neuropharmacology. 2012;63(1):97-110. DOI 10.1016/
j.neuropharm.2012.04.017

Mustafin Z.S., Lashin S.A., Matushkin Yu.G. Phylostratigraphic
analysis of gene networks of human diseases. Vavilovskii Zhurnal
Genetiki i Selektsii = Vavilov Journal of Genetics and Breeding.
2021;25(1):46-56. DOI 10.18699/VJ21.006

New D.C., Wong J.T. The evidence for G-protein-coupled receptors
and heterotrimeric G proteins in protozoa and ancestral metazoa.
Biol. Signals Recept. 1998;7(2):98-108. DOI 10.1159/000014535

Olszewski P.K., Cedernaes J., Olsson F., Levine A.S., Schiöth H.B.
Analysis of the network of feeding neuroregulators using the Allen
Brain Atlas. Neurosci. Biobehav. Rev. 2008;32(5):945-956. DOI
10.1016/j.neubiorev.2008.01.007

Pandy-Szekeres G., Caroli J., Mamyrbekov A., Kermani A.A., Keseru
G.M., Kooistra A.J., Gloriam D.E. GPCRdb in 2023: state-specific
structure models using AlphaFold2 and new ligand resources.
Nucleic Acids Res. 2023;51(D1):D395-D402. DOI 10.1093/nar/
gkac1013

Rebello C.J., Greenway F.L. Reward-induced eating: therapeutic approaches
to addressing food cravings. Adv. Ther. 2016;33(11):1853-
1866. DOI 10.1007/s12325-016-0414-6

Sarnat H.B., Netsky M.G. When does a ganglion become a brain? Evolutionary
origin of the central nervous system. Semin. Pediatr. Neurol.
2002;9(4):240-253. DOI 10.1053/spen.2002.32502

Siegal E., Hooker S.K., Isojunno S., Miller P.J.O. Beaked whales and
state-dependent decision-making: how does body condition affect
the trade-off between foraging and predator avoidance? Proc. Biol.
Sci. 2022;289(1967):20212539. DOI 10.1098/rspb.2021.2539

Spetter M.S., de Graaf C., Mars M., Viergever M.A., Smeets P.A.
The sum of its parts – effects of gastric distention, nutrient content
and sensory stimulation on brain activation. PLoS One. 2014;9(3):
e90872. DOI 10.1371/journal.pone.0090872

Tomé D., Schwarz J., Darcel N., Fromentin G. Protein, amino acids,
vagus nerve signaling, and the brain. Am. J. Clin. Nutr. 2009;90(3):
838S-843S. DOI 10.3945/ajcn.2009.27462W

Tremblay A., Bellisle F. Nutrients, satiety, and control of energy intake.
Appl. Physiol. Nutr. Metab. 2015;40(10):971-979. DOI 10.1139/
apnm-2014-0549

Ward A.E., Rosenthal B.M. Evolutionary responses of innate immunity
to adaptive immunity. Infect. Genet. Evol. 2014;21:492-496. DOI
10.1016/j.meegid.2013.12.021

Wells A., Kopp N., Xu X., O’Brien D.R., Yang W., Nehorai A., Adair-
Kirk T.L., Kopan R., Dougherty J.D. The anatomical distribution of
genetic associations. Nucleic Acids Res. 2015;43(22):10804-10820.
DOI 10.1093/nar/gkv1262

Yang D., Zhou Q., Labroska V., Qin S., Darbalaei S., Wu Y., Yuliantie
E., Xie L., Tao H., Cheng J., Liu Q., Zhao S., Shui W., Jiang Y.,
Wang M.W. G protein-coupled receptors: structure- and functionbased
drug discovery. Signal Transduct. Target. Ther. 2021;6(1):7.
DOI 10.1038/s41392-020-00435-w

Yeo G.S., Heisler L.K. Unraveling the brain regulation of appetite: lessons
from genetics. Nat. Neurosci. 2012;15(10):1343-1349. DOI
10.1038/nn.3211

Zhang L.L., Wang J.J., Liu Y., Lu X.B., Kuang Y., Wan Y.H., Chen Y.,
Yan H.M., Fei J., Wang Z.G. GPR26-deficient mice display increased
anxiety- and depression-like behaviors accompanied by
reduced phosphorylated cyclic AMP responsive element-binding
protein level in central amygdala. Neuroscience. 2011;196:203-214.
DOI 10.1016/j.neuroscience.2011.08.069

